# Analysis of gene expression profiles in Alzheimer’s disease patients with different lifespan: A bioinformatics study focusing on the disease heterogeneity

**DOI:** 10.3389/fnagi.2023.1072184

**Published:** 2023-02-23

**Authors:** Ji Zhang, Xiaojia Li, Jun Xiao, Yang Xiang, Fang Ye

**Affiliations:** ^1^Department of Neurology, West China Hospital of Sichuan University, Chengdu, China; ^2^Department of Neurology, Sichuan Academy of Medical Sciences & Sichuan Provincial People’s Hospital, Chengdu, China; ^3^Department of Neurology, Chinese Academy of Sciences Sichuan Translational Medicine Research Hospital, Chengdu, China

**Keywords:** Alzheimer’s disease, lifespan, differentially expressed genes, functional enrichment analysis, hub gene

## Abstract

**Objective:**

Alzheimer’s disease (AD) as the most frequent neurodegenerative disease is featured by gradual decline of cognition and social function in the elderly. However, there have been few studies focusing on AD heterogeneity which exists both genetically and clinically, leading to the difficulties of AD researches. As one major kind of clinical heterogeneity, the lifespan of AD patients varies significantly. Aiming to investigate the potential driving factors, the current research identified the differentially expressed genes (DEGs) between longer-lived AD patients and shorter-lived ones *via* bioinformatics analyses.

**Methods:**

Qualified datasets of gene expression profiles were identified in National Center of Biotechnology Information Gene Expression Omnibus (NCBI-GEO). The data of the temporal lobes of patients above 60 years old were used. Two groups were divided according to the lifespan: the group ≥85 years old and the group <85 years old. Then GEO2R online software and R package of Robust Rank Aggregation (RRA) were used to screen DEGs. Bioinformatic tools were adopted to identify possible pathways and construct protein–protein interaction network.

**Result:**

Sixty-seven AD cases from four qualified datasets (GSE28146, GSE5281, GSE48350, and GSE36980) were included in this study. 740 DEGs were identified with 361 upregulated and 379 downregulated when compared longer-lived AD patients with shorter-lived ones. These DEGs were primarily involved in the pathways directly or indirectly associated with the regulation of neuroinflammation and cancer pathogenesis, as shown by pathway enrichment analysis. Among the DEGs, the top 15 hub genes were identified from the PPI network. Notably, the same bioinformatic procedures were conducted in 62 non-AD individuals (serving as controls of AD patients in the four included studies) with distinctly different findings from AD patients, indicating different regulatory mechanisms of lifespan between non-AD controls and AD, reconfirming the necessity of the present study.

**Conclusion:**

These results shed some lights on lifespan-related regulatory mechanisms in AD patients, which also indicated that AD heterogeneity should be more taken into account in future investigations.

## 1. Introduction

Alzheimer’s disease (AD), featured by progressive decline of cognition and individual social functioning, is the most prevalent neurodegenerative disease in older people (Scheltens et al., 2016). AD accounts for more than half of all dementia cases, leading to serious burdens on the patients, the families and the society as a whole (Jia et al., 2018). The typical pathological characteristics of AD were recognized to be hyper-phosphorylated tau aggregations and amyloid-β (Aβ) plaques in the brain (Bakota and Brandt, 2016). However, it has been well aware that Aβ pathology and tau pathology could not represent the whole picture of the pathogenesis of AD. Thus, researchers have developed more hypotheses hoping to clarify its pathogenesis, such as neuroinflammation, oxidative stress and mitochondrial dysfunction, protein oxidation, lipid peroxidation, etc. (Serrano-Pozo et al., 2011). However, the exact mechanisms leading to the beginning and development of AD still need to be further clarified.

One major reason might be the huge heterogeneity of AD, both genetically and clinically (Devi and Scheltens, 2018). It has long been acknowledged that the clinical manifestations of AD patients vary significantly in many aspects including but not limited to the onset age, progressive rate, the lifespan, the affected cognitive domains, and so on(Lam et al., 2013). Thanks to the uncovering of many AD risk genes using high-throughput biochips in recent decades, AD has been recognized to be the dysregulation of a substantial number of genes resulting in the alteration of their complex interactions, which finally leads to the varieties of disease manifestations (Zhu et al., 2017). Some previous studies have focused on the link between its genetic and clinical heterogeneity with results suggesting that using more genetically or clinically homogeneous patients may be helpful to identify additional risk genes. Lo et al.’s (2019) study performed stratified gene-based genome-wide association studies (GWAS) and polygenic variation analyses in the younger and older age-at-onset groups in order to explore genetic heterogeneity of AD related to age and locate risk genes showing different effects across age. Belloy et al. (2020) probe the link between longevity gene KLOTHO and the APOE4-AD risk and found that KL-VS (a functional variant of KLOTHO) heterozygosity was significantly associated with decreased risk for AD and conversion to AD, and also reduced Aβ biomarkers in individuals who carry APOE4 but not in those who do not carry APOE4. These results suggest that there might be different regulatory mechanisms in different AD subgroups, which are of great significance to be further investigated. However, AD was studied as a monolithic disease in most studies and compared with non-AD controls, which might cause considerable confounding when exploring its pathogenesis.

Notably, the lifespan of AD patients also exhibits considerable heterogeneity. Some AD patients present with later onset and/or slower progression leading to longer lifespan, while some others might have significantly shorter lifespan. Although one of the major targets of AD intervention is to prolong patients’ lifespan, the heterogeneity in AD lifespan has not been much explored. Aoyagi et al.’s (2019) study quantically measures the intracellular self-propagating conformers in postmortem brain samples from AD patients and shows that the longevity-dependent reduction in self-propagating tau conformers were identified in spite of increasing levels of total insoluble tau, demonstrating an inverse correlation between longevity and the amounts of pathological tau conformers in AD patients. The underlying mechanisms have not been clarified so far. In this case, analyzing lifespan-related gene expression profiles in AD patients might be a promising strategy to provide information about the genetic regulatory mechanisms underlying the phenotype of different lifespans. To date, there has been no such study published before.

This study acquired qualified gene profiles of AD patients from GEO database and the differentially expressed genes (DEGs) between AD patients with longer lifespan and shorter lifespan were meta-analyzed using the R package of Robust Rank Aggregation (RRA). Then, the functional pathway annotations and protein–protein interaction (PPI) networks of DEGs were performed *via* bioinformatics approaches. We investigate the lifespan-related regulatory mechanisms in AD patients at a molecular level and help uncovering potential candidate genes for AD intervention.

## 2. Methods

### 2.1. Dataset selection and data preprocessing

**Figure 1:**
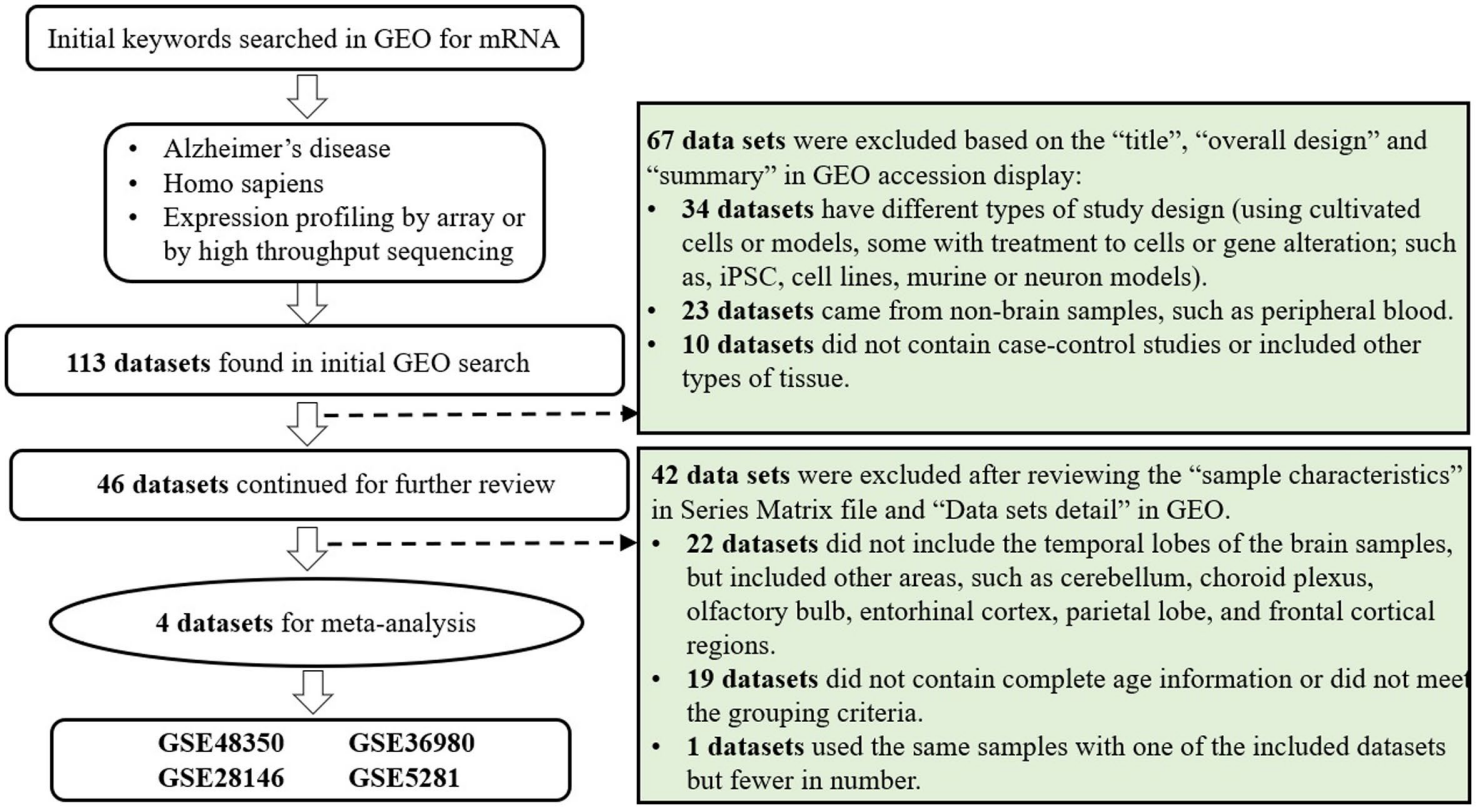
Data set selection flowchart.

The Gene Expression Omnibus (GEO)^1^ is a public repository for researchers worldwide to submit high-throughput microarray and next-generation sequence functional genomic datasets. All data are available for download without charge (Barrett et al., 2013). The datasets of gene expression profiles used in the present study were obtained from GEO with the search strategy as follows: (((Expression profiling by high throughput sequencing [DataSet Type]) OR Expression profiling by array [DataSet Type]) AND homo sapiens[Organism]) AND Alzheimer’s disease[Title] (Figure 1). The inclusion criteria of qualified datasets were as follows: investigating the expression profiles by arrays or high throughput sequencing in GEO; using brain samples of AD cases and non-AD controls; containing complete information of age at death. Since the brain samples were donated by volunteers and collected postmortem, the ages displayed in these studies were in fact the ages at death, serving as a qualified indicator of lifespan.

Through literature reviewing, it was found that several datasets (for example GSE48350, GSE5281, GSE36980) are designed to obtain samples from multiple brain regions of one donor. However, one previous study(Moradifard et al., 2018) has proved that the gene expression profiles vary across different brain regions. Thus, it might cause substantial confounding if all the samples were included in the meta-analysis. Thus, only the samples of temporal lobe were chosen for the analysis in order to minimize the heterogenicity of samples. If one dataset included samples of different regions in the temporal lobe, the region with the largest sample size was chosen. With regard to the cut-off age, it was firstly set to be above 80 years old which has been reported to be the average life expectancy of Chinese elderly (Huang et al., 2021). After several grouping attempts, the cut-off age of 85 years old was selected which would include more datasets and make the grouping more balanced. Then, according to the lifespan: the group with longer lifespan (> = 85 years old) and the group with shorter lifespan (<85 years old), the samples of each dataset were divided into two groups. In addition, the samples with age over 60 years old were chosen to lower the possible influences of unnatural deaths.

### 2.2. DEGs identification

The R tools GEOquery and limma from the Bioconductor project were used to export and analyze the gene expression data of the comparisons between AD patients (AD patients with longer lifespan vs. those with shorter lifespan). Bioconductor, an open-source software project built on the R programming language, offers tools for the study of high-throughput genetic data. The R package GEOquery transforms GEO data into R data structures for usage by other R tools (Davis and Meltzer, 2007). Differentially expressed genes (DEGs) between the two groups of each dataset with *p* values <0.05 were selected to be further analyzed. Then the values of fold changes (FC) were log2 transformed and represented as log FC in short. Log FCs which were below zero indicated the DEGs were down regulated, and vice versa. The meta *p* values of the DEGs were calculated using the R package of Robust Rank Aggregation (RRA) and the results were represented as meta-analysis scores (Kolde et al., 2012). The RRA technique, which can manage fluctuating gene content from various microarray platforms in the presence of noise or with partial rankings, is based on a comparison of real data with a null model that assumes random order of input lists. Besides, the mean values of log FCs were also calculated. Genes with meta *p* values less than 0.05 and average |log FC| ≥ 1 were considered as final DEGs. Data processing was performed using Python Jupyter Notebook (Edition 5.0.0).

Notably, data of non-AD controls were also analyzed using the same methodology to serve as comparisons. The non-AD data came from the included datasets and were used to be controls of AD patients in the original studies.

### 2.3. Gene functional enrichment analysis

The DEGs were uploaded to Metascape^2^ (Zhou et al., 2019). Pathway and process enrichment analyses were carried out with ontology sources of KEGG pathway, GO Biological Processes, Reactome Gene Sets, Canonical Pathways, and WikiPathways. Genes of the whole genome were adopted as the enrichment background. Terms with *p* value <0.01, count of genes ≥3, and an enrichment factor > 1.5 were collected and grouped into clusters based on their membership similarities. The top 20 clusters were collected using the most statistically significant term in each cluster as the representative.

Protein–protein interaction (PPI) enrichment analysis was conducted based on the following databases: STRING, BioGrid, OmniPath, InWeb_IM. If the network contains 3 to 500 proteins, the Molecular Complex Detection (MCODE) algorithm would be applied to identify densely connected network components. Pathway and process enrichment analysis was applied to each MCODE component independently, and the three best-scoring terms by value of *p* were retained as the functional description of the corresponding components.

### 2.4. Hub genes identification and association enrichment analysis

To screen hub genes, CytoHubba plug-in of Cytoscape was utilized to analyze PPI networks exported from the corresponding Metascape results in the present study (Jeong et al., 2001). The top 15 hub genes ranked by the method of Maximal Clique Centrality (MCC) were calculated. Enrichment analysis were also performed in ontology categories of DisGeNET *via* Metascape (Piñero et al., 2017). DisGeNET integrates data from expert curated repositories, GWAS catalogs, animal models and the scientific literature to provide information about the genetic basis of human diseases. Genes of the whole genome were adopted as the enrichment background. Terms with *p* value <0.01, count of genes ≥3, and an enrichment factor > 1.5 were collected and grouped into clusters based on their membership similarities.

### 2.5. Analysis of immune infiltration and hub genes

The gene sets of 28 immune cells and four classes of immune factors were downloaded from TISIDB database.^3^ The following 28 types of immune cells were obtained: central memory CD4+ T cells (CD4+ Tcm), central memory CD8+ T cells (CD8+ Tcm), type-2 T helper cells (Th2), CD56dim natural killer cells (CD56− NK), activated CD8+ T cells (CD8+ Ta), activated CD4+ T cells (CD4+ Ta), activated B cells (Ba), effector memory CD8+ T cells (CD8+ Tem), effector memory CD4+ T cells (CD4+ Tem), macrophages, eosinophils, memory B cells (Bm), immature dendritic cells (DCi), gamma delta T cells (γδT), CD56bright natural killer cells (CD56+ NK), monocytes, mast cells, natural killer cells (NK), immature B cells (Bi), type-1 T helper cells (Th1), neutrophils, plasmacytoid dendritic cells (DCp), natural killer T cells (NK T), type-17 T helper cells (Th17), follicular helper T cells (Tfh), regulatory T cells (Tregs), myeloid-derived suppressor cells (MDSC), and activated dendritic cells (DCa). The four classes of immune factors include 41 chemokines, 24 immunosuppressive factors, 46 immunostimulatory factors, and 18 immune receptors.

The ssGSEA algorithm, which classifies gene sets with common biological functions, physiological regulation, and chromosomal localization, was employed *via* R packages (GSVA 1.42.0) to comprehensively assess the immunologic characteristics of each sample included in the analyses (Hänzelmann et al., 2013). Normalized data of gene expression profiles were compared with the gene sets to demonstrate the enrichment of immune cells in each AD brain samples. Then, ANOVA was adopted to identify immune cell types with significant differences between the groups with longer lifespan and shorter lifespan. Pearson correlations between the gene expression level of each hub gene and the concentrations of immune cells were carried out using cor.test in R software (version: 4.0.3). The hub genes were identified in 2.4.

The correlations between the gene expression levels of each hub gene and the gene sets of immune factors were also calculated, respectively. Then, the pairs of hub genes and immune-related molecules with |cor| > 0.6 & *p* value<0.05 were selected to generate a circos plot *via* Cytoscape.

## 3. Result

### 3.1. Identification of DEGs

The flowchart of dataset selection was shown in Figure 1. Four qualified microarray datasets (GSE48350, GSE5281, GSE28146, GSE36980) and one dataset of high throughput sequencing (GSE173955) were identified according to the inclusion and grouping criteria. Thereinto, the samples used in GSE173955 were also used in GSE36980 as stated in the abstract of the article (Mizuno et al., 2021). In order to include more samples and reduce batch effect and other confounding, GSE36980 were included in the analysis rather than including both or GSE173955 alone.

**Figure 2:**
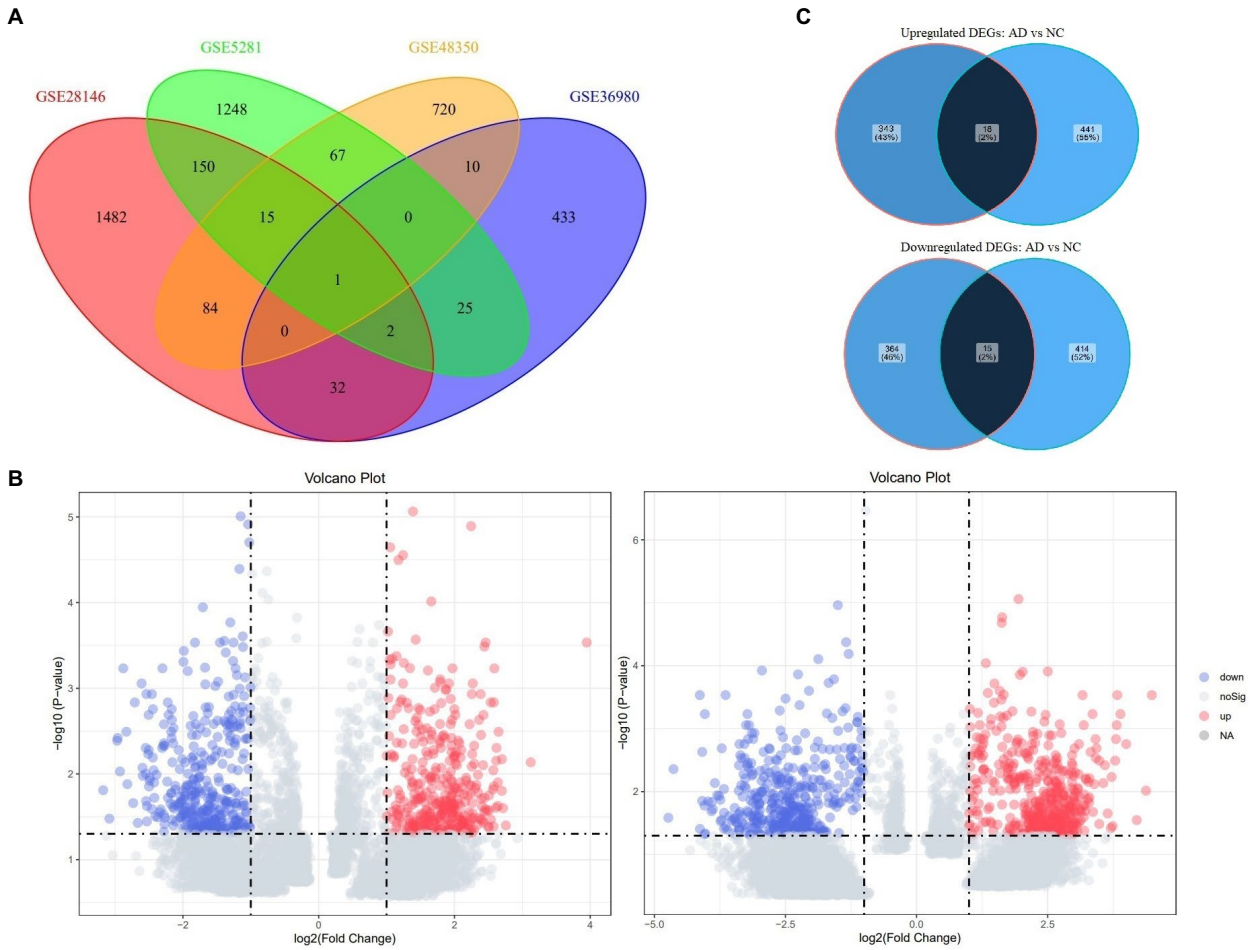
Venn diagrams of Four datasets and Volcano plots of DEGs in the groups of AD and non-AD controls. **(A)** Venn diagram of the overlap of genes lists from the Four included datasets. **(B)** Volcano plots of DEGs in the groups of AD (left) and non-AD controls (right). **(C)** Venn diagram of the overlap of DEGs between the groups of AD and non-AD controls. AD, Alzheimer’s disease; DEG, differentially expressed genes.

In total, 129 samples (62 non-AD controls and 67 AD cases) were analyzed in this study; the grouping and baseline information were shown in Table 1. After comparing longer-lived AD patients with shorter-lived ones in each dataset, genes with *p* < 0.05 were selected and formed a list, respectively. The Venn diagram showing the overlap of the four gene lists was displayed as Figure 2A. After meta-analysis, a list of 740 DEGs with 361 upregulated and 379 downregulated was identified in the AD group with longer lifespan compared to that with shorter lifespan. The top 15 most significantly upregulated and downregulated genes when comparing longer-lived individuals with shorter-lived ones in AD patients were shown in Table 2.

**Table 1:** Characteristics of the individual studies.

GEO ID	Year	Location and array platform	Brain region	AD samples						Non-AD samples					
				Long lifespan (> = 85 years old)			Short lifespan (<85 years old)			Long lifespan > = 85 years old			Short lifespan <85 years old		
				No	Male	Age	No	Male	Age	No	Male	Age	No	Male	Age
GSE48350	2014	USA GPL570	Hippocampus	11	5	88.91 ± 3.18	8	4	75.00 ± 5.98	10	5	91.80 ± 4.60	15	8	75.40 ± 6.17
GSE5281	2006	USA GPL570	Medial temporal gyrus	4	2	87.50 ± 1.80	12	8	76.33 ± 4.29	3	1	91.67 ± 7.41	9	7	76.22 ± 6.23
GSE28146	2011	USA GPL570	CA1 gray matter	13	4	91.15 ± 4.70	9	2	79.22 ± 5.27	5	3	91.20 ± 4.58	3	3	78.33 ± 2.36
GSE36980	2013	Japan GPL6244	Temporal gyrus	8	3	91.38 ± 2.96	2	2	83.50 ± 0.50	4	2	87.75 ± 1.30	13	5	76.77 ± 5.16

**Table 2:** Top 15 differentially expressed genes (DEGs) identified in the meta-analysis comparing the longer-lived AD group with the shorter-lived one.

Up-regulated			Down-regulated		
Gene symbols	Average Log (FC)	Meta-analysis score	Gene symbols	Average Log (FC)	Meta-analysis score
RNMT	1.388641	8.63E-06	ACAN	−1.14943	9.83E-06
ZBED3-AS1	2.246284	1.28E-05	TNRC6C	−1.03933	1.22E-05
POMZP3	1.242601	2.79E-05	ST3GAL4-AS1	−1.02212	1.98E-05
L3MBTL1	1.175608	3.18E-05	FBXL17	−1.1674	4.05E-05
FBLIM1	1.659288	9.69E-05	KLK8	−1.70629	0.0001
DRICH1	1.021555	0.0002	SUCLG2-AS1	−1.3024	0.0001
SLC44A5	1.429692	0.0002	GAS2L3	−1.11936	0.0002
EZH2	2.459765	0.0002	PKNOX1	−1.39156	0.0003
PLN	3.948696	0.0002	GNRH1	−1.45433	0.0003
PVALB	2.440934	0.0003	NR0B1	−1.8225	0.0003
DYNC1H1	1.146785	0.0004	ZNF366	−1.25511	0.0003
LRRC28	1.081166	0.0005	IL17RB	−1.1153	0.0003
EBP	1.045227	0.0005	RBM33	−1.98674	0.0004
UHRF1BP1L	1.254153	0.0005	PACSIN2	−1.3685	0.0004
ZNF81	1.057144	0.0005	AREG	−1.26	0.0005

Ave log (FC), average log2 fold-change.

In addition, the data of non-AD controls were also analyzed using the same methodology to serve as comparison and 888 DEGs were identified with 459 up-regulated and 429 down-regulated. Volcano plots showing DEGs from both comparisons (the groups of AD and non-AD controls) were as Figure 2B. The Venn diagrams showing the overlap of AD and non-AD DEGs were exhibited in Figure 2C.

### 3.2. Gene functional enrichment analysis of DEGs and hub genes identification

**Figure 3:**
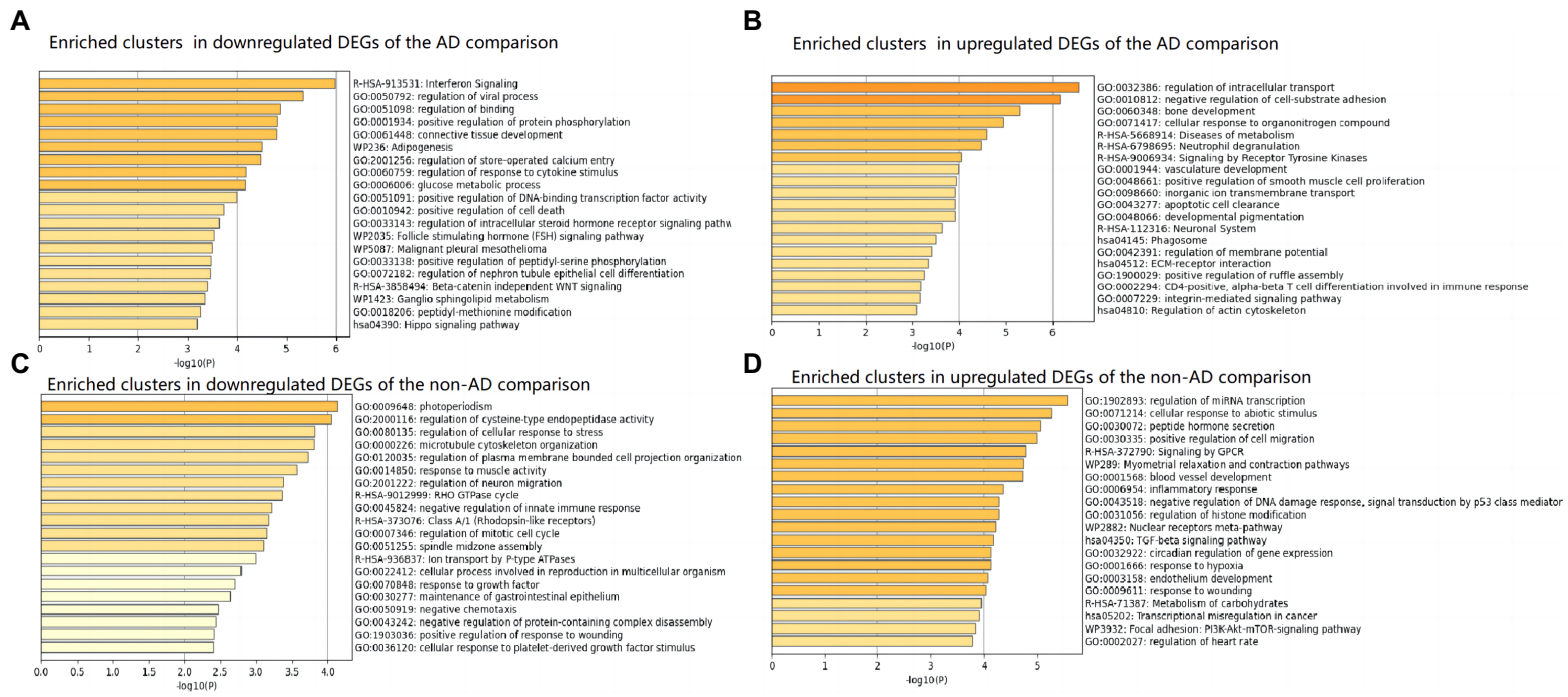
Top 20 clusters with their representative enriched terms (one per cluster) in up-and downregulated DEGs of AD and non-AD comparisons, respectively. **(A)** Enriched clusters in downregulated DEGs of the AD comparison. **(B)** Enriched clusters in upregulated DEGs of the AD comparison. **(C)** Enriched clusters in downregulated DEGs of the non-AD comparison. **(D)** Enriched clusters in upregulated DEGs of the non-AD comparison. AD, Alzheimer’s disease; DEG, differentially expressed genes.

**Figure 4:**
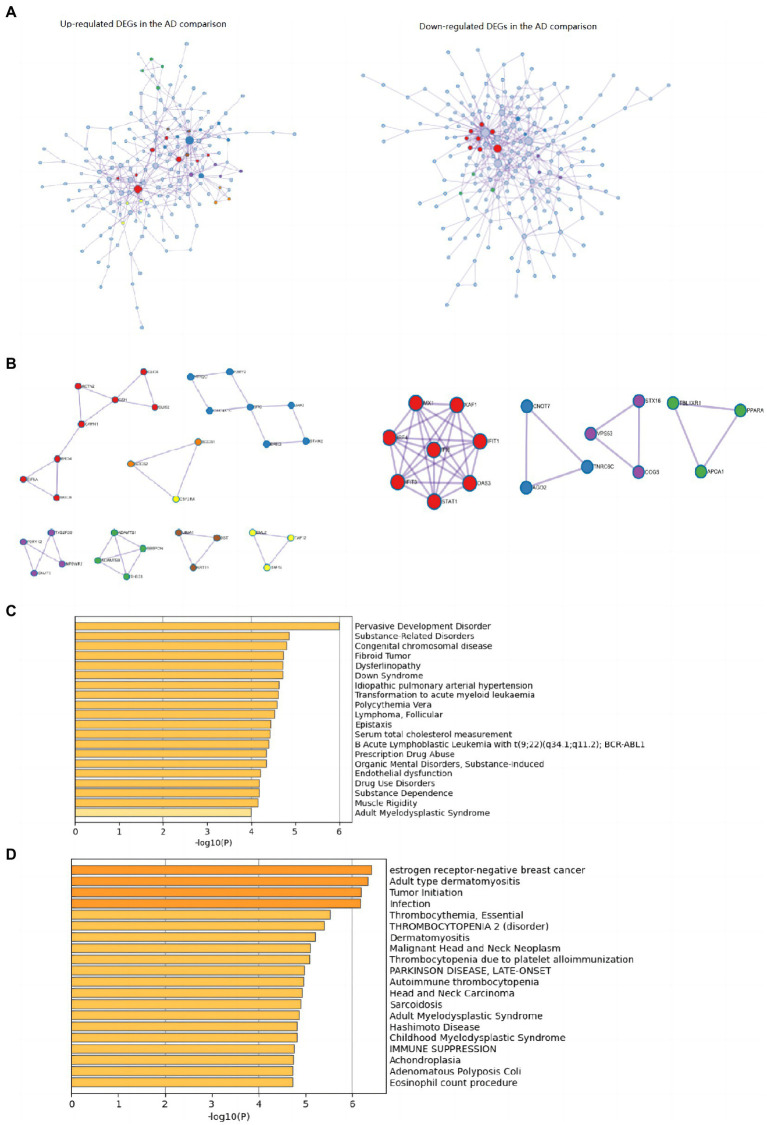
PPI networks and top 20 clusters enriched in DisGeNET in up-and downregulated DEGs of AD comparison. **(A)** PPI networks identified in the DEGs of AD comparison (Left: upregulated; right: downregulated). **(B)** MCODE components identified in the DEGs of AD comparison (Left: upregulated; right: downregulated). **(C)** Top 20 clusters enriched in DisGeNET in upregulated DEGs of AD comparison. **(D)** Top 20 clusters enriched in DisGeNET in downregulated DEGs of AD comparison. AD, Alzheimer’s disease; DEG, differentially expressed genes.

The top 20 clusters with their representative enriched terms (one per cluster) of the up-and downregulated DEGs in the AD and non-AD comparisons were displayed in Figure 3. More details of the top five clusters were shown in Tables 3, 4. The PPI networks and MCODE components identified in the DEGs of the AD comparison were shown in Figures 4A,B. The top clusters (one term per cluster) of enrichment analysis in DisGeNET were shown in Figure 4C.

**Table 3:** The top 5 clusters with their representative enriched terms (one per cluster) of the upregulated DEGs in AD group with longer lifespan.

GO	Category	Description	Count	Log10(P)	Gene Hits
GO:0032386	GO Biological Processes	Regulation of intracellular transport	17	−6.55	ACTN2|CD36|DYNC1H1|STOM|GAS1|JAK2|NF1|PLN|SRC|ITGB
					1BP1|CAPN10|DNAJC13|NRDE2|RIOK2|MAVS|SH3TC2|HPS4
GO:0010812	GO Biological Processes	Negative regulation of cell-substrate adhesion	8	−6.15	ANGPT2|BCL6|COL1A1|EFNA5|NF1|SRC|THBS1|ITGB1BP1
GO:0060348	GO Biological Processes	Bone development	12	−5.29	BGN|COL1A1|RARA|SHOX2|SRC|FGF18|EBP|FOXP1|PDGFC|
					TMEM107|NOTUM|FREM1
GO:0071417	GO Biological Processes	Cellular response to organonitrogen compound	20	−4.93	ACTN2|CD36|CHRM4|COL1A1|COL4A1|CSK|EZH2|HTR2C|JAK
					2|P2RY2|PDE3A|SRC|SOCS1|SOCS2|BCL2L11|RRAGB|CAPN10|
R-HSA-5668914	Reactome Gene Sets	Diseases of metabolism	12	−4.59	BGN|CSF2RA|SLC37A4|HLCS|MGAT2|MUC7|THBS1|CUBN|
					ADAMTS1|ADAMTS9|ALG13|SBSPON

GO, gene ontology; BP, biological process. Count is the number of genes in the user-provided lists with membership in the given ontology term. “Log10(P)” is the *p*-value in log base 10.

**Table 4:** The top 5 clusters with their representative enriched terms (one per cluster) of the downregulated DEGs in AD group with longer lifespan.

GO	Category	Description	Count	Log10(P)	Gene Hits
R-HSA-913531	Reactome Gene Sets	Interferon Signaling	13	−5.98	CD44|IFI6|HLA-DRB4|IFIT1|IFIT3|IRF4|KPNA4|MX1|
					OAS3|STAT1|NUP210|RIGI|XAF1
GO:0050792	GO Biological Processes	Regulation of viral process	11	−5.33	NR5A2|GSN|IFIT1|MX1|OAS3|PPARA|SLPI|
					STAT1|CXCR4|HMGA2|CNOT7
GO:0051098	GO Biological Processes	Regulation of binding	16	−4.87	BDNF|HFE|IFIT1|IRF4|PPARA|SLPI|STK4|HMGA2|SYMPK|
					ADAM15|MBD2|HIPK2|TRIB3|ARHGAP28|PARP9|SPPL3
GO:0001934	GO Biological Processes	Positive regulation of protein phosphorylation	24	−4.80	AREG|BDNF|BMP3|CD44|CKS2|HFE|IL6|ITGB3|LTK|PTGS2|
					STK4|HMGA2|FZD1|TNFRSF10B|GPRC5A|MAP3K13|
					TCL1B|HDAC6|HIPK2|ALS2|CLSPN|PARP9|PROM2|CD24
GO:0061448	GO Biological Processes	Connective tissue development	12	−4.79	BMP1|BMP3|CD44|EVC|HOXA5|LTBP3|MGP|
					HMGA2|TRIP11|RASAL2|CREB3L2|TBL1XR1

GO, gene ontology; BP, biological process. Count is the number of genes in the user-provided lists with membership in the given ontology term. “Log10(P)” is the *p*-value in log base 10.

When comparing AD patients with longer lifespan to those with shorter lifespan, the three best-scoring terms identified *via* pathway and process enrichment analysis to each MCODE component were as follows: cellular response to nitrogen compound (GO: 1901699, Log10(P) = −7.9), cellular response to organonitrogen compound (GO: 0071417, Log10(P) = −7.9) and regulation of intracellular transport (GO:0032386, Log10(P) = −7.3) in the upregulated DEGs; Interferon Signaling (R-HSA-913531, Log10(P) = −7.6), regulation of viral process (GO:0050792, Log10(P) = −7.6), Interferon alpha/beta signaling (R-HSA-909733, Log10(P) = −7.3) in the downregulated DEGs.

The top 15 hub genes identified in the PPI network of the up-regulated DEGs were SRC (MCC score = 44), RPL24 (MCC score = 33), BRD4 (MCC score = 32), RPL10L (MCC score = 30), CSK (MCC score = 22), JAK2 (MCC score = 20), MRPL4 (MCC score = 20), UBD (MCC score = 19), EIF5A (MCC score = 18), WDR61 (MCC score = 16), CLUH (MCC score = 16), EZH2 (MCC score = 15), CAPN1 (MCC score = 13), ACTN2 (MCC score = 13), CLIC2 (MCC score = 12), in order of ranks. The top 15 hub genes identified in the PPI network of the downregulated DEGs were STAT1 (MCC score = 5,079), MX1 (MCC score = 5,066), IFIT3 (MCC score = 5,064), IFIT1 (MCC score = 5,064), OAS3 (MCC score = 5,043), IRF4 (MCC score = 5,043), XAF1 (MCC score = 5,043), IFI6 (MCC score = 5,040), DDX58 (MCC score = 65), HDAC6 (MCC score = 25), RSL1D1 (MCC score = 24), BIRC3 (MCC score = 22), RPS6 (MCC score = 21), BRD7 (MCC score = 14), RRP12 (MCC score = 14), in order of ranks.

### 3.3. Analysis of immune infiltration and hub genes

**Figure 5:**
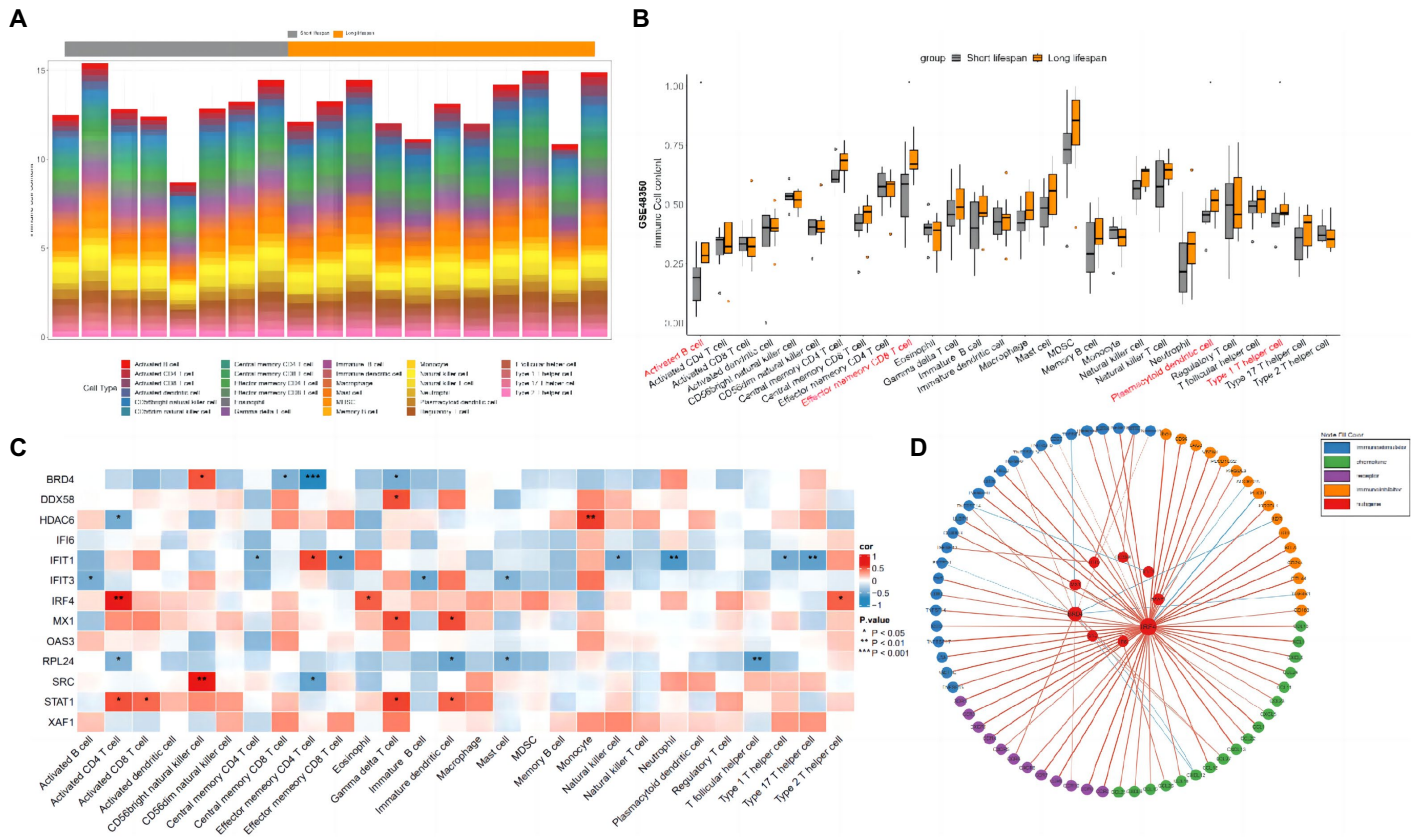
Immune infiltration analysis between longer-lived AD and shorter-lived ones. **(A)** The column diagram displaying the relative percentage of the 28 immune cells between in AD samples. **(B)** The difference of immune infiltration between longer-live AD (orange) and shorter-lived ones (gray; * indicates *p*-values < 0.05). **(C)** Correlations between hub genes and the infiltration levels of the 28 immune cells. **(D)** Circos plot of the interactions between the hub genes and immune-related molecules.

The gene expression profiles of GSE48350 samples (Table 1) were used to perform immune infiltration analysis. As shown in Figures 5A,B, the fractions for activated B cell, effector memory CD8 T cell, plasmacytoid dendritic cell and type 1 T helper cell in the longer-lived AD group were remarkably higher than in those of shorter-lived ones.

Since most pathways identified in the downregulated DEGs were inflammation related, the top 10 hub genes identified in the downregulated DEGs and the top 3 hub genes identified in the upregulated DEGs were selected for the association analysis with immune cells and immune factors. As shown in Figure 5C, STAT1 was positively correlated with gamma delta T cell, activated CD4 T cell, immature dendritic cell and activated CD8 T cell. MX1 was positively correlated with immature dendritic cell and gamma delta T cell. IFIT3 was negatively correlated with immature B cell, activated B cell and mast cell. IFIT1 was positively correlated with effector memory CD4 T cell and negatively correlated with neutrophil, type 17 T helper cell, effector memory CD8 T cell, natural killer cell, type 1 T helper cell and central memory CD8 T cell. IRF4 was positively correlated with activated CD4 T cell, eosinophil and type 2 T helper cell. DDX58 was positively correlated with gamma delta T cell. HDAC6 was positively correlated with monocyte. SRC was positively correlated with CD56 bright natural killer cell and negatively correlated with effector memory CD4 T cell. RPL24 was negatively correlated with T follicular helper cell, immature dendritic cell, mast cell and activated CD4 T cell. BRD4 was positively correlated with CD56 bright natural killer cell and negatively correlated with effector memory CD4 T cell, gamma delta T cell and central memory CD8 T cell. There were no significant findings when analyzing the associations between immune cells and the remaining hub genes (OAS3, XAF1, and IFI6). Protein–protein interaction plot of hub genes and immune-related molecules was shown as Figure 5D.

## 4. Discussion

In the present study, 740 DEGs with 361 upregulated and 379 downregulated were identified comparing AD patients with longer lifespan to those with shorter lifespan. Bioinformatic analyses were performed based on these DEGs, and the significant findings would be discussed as below. Notably, the same bioinformatic procedures and analyses were conducted basing on the data of non-AD controls (Table 1), with distinctly different findings from those identified in the AD comparison (Figures 2C, 3). These results indicated that the underlying regulatory mechanisms of AD lifespan might be quite different from those of non-AD controls, reconfirming the necessity of the present study. Investigating lifespan-related gene expression profiles in AD patients would help to understand the genetic background possibly impacting its clinical course, which has not been published before.

In the lifespan-related pathways identified in the present study, multiple clusters of pathways were directly or indirectly associated to neuroinflammation. The directly associated clusters included those represented by the pathways of interferon Signaling (R-HSA-913531) and regulation of response to cytokine stimulus (GO:0060759) in the downregulated DEGs. The indirectly associated clusters included those about antiviral responses represented by the pathway of regulation of viral process in the downregulated DEGs; those about metabolism processes represented by the pathways of Adipogenesis, glucose metabolic process in the downregulated DEGs; Diseases of metabolism in the upregulated DEGs; and those about autophagy represented by the pathways of apoptotic cell clearance, Phagosome in the upregulated DEGs. These results indicated that neuroinflammation might be closely related to the regulation of AD lifespan.

Amounts of evidence, involving increasing numbers of activated microglial and astroglia in the brains of AD patients, elevated pro-inflammatory cytokine in AD brains, and epidemiological proof that chronic non-steroidal anti-inflammatory drug used before AD associates to a lower incidence, have suggested that neuroinflammation, an early-emerging and continuously existing feature of AD, plays a significant part in the pathogenesis of the disorder (Calsolaro and Edison, 2016). Interferons (IFNs) are a superfamily of cytokine proteins that play a significant part in host immune response to pathogens, infections, and various diseases (de Weerd and Nguyen, 2012). It has been proved that they are critical in the exacerbation of neuroinflammation and actively contribute to AD progression (Taylor et al., 2018). Also, studies have shown that active virus infections in brain may not only accelerate amyloid deposition and the progression of AD (Eimer et al., 2018; Mangold and Szpara, 2019), but also, by inhibiting autophagy, disrupt clearance of the aberrant proteins, resulting in their accumulation and deposition, and finally to AD onset and progression (Itzhaki, 2017). dysregulation of metabolism processes would lead to metabolic changes, induction of obesity and adipose tissue inflammation, resulting in the acceleration of systemic low-grade inflammation and then accumulation of toxic amyloid, eventually the onset of AD (Więckowska-Gacek et al., 2021). Regulation of these pathways might result in the mitigation of excessive neuroinflammation in AD brains and thus leading to longer lifespan. In addition, the results of immune infiltration analysis also supported this conclusion, which showed that four kinds of immune cells increased significantly in longer-lifespan AD patients and the hub genes corelated with multiple immune cells and immune factors, indicating that the regulation of AD lifespan might be intertwined with the complex networks of neuroinflammation.

Thus, identifying key mediators regulating the neuroinflammation process might be helpful to develop anti-inflammatory therapies for AD (Taylor et al., 2018). Among the identified hub genes, STAT1, which ranked the first in the hub gene list identified in the downregulated DEGs and corelated with multiple immune cells and immune factors, has already come into notice of researchers. The protein encoded by STAT1 is activated by varieties of ligands including IFN-α, EGF, IFN-γ, PDGF, and IL6. Zhang et al.’s (2021) study shows that STAT1 knockout suppresses AD typical pathologies. Another study identifies that STAT1 activation abolishes expression of N-methyl-D-aspartate receptors (NMDARs), while the downregulation of STAT1 efficiently mitigates Tau-induced suppression of NMDAR expression and improves the function of synapses and performances in memory tests (Li et al., 2019). He et al.’s (2021) study shows that the overexpression of STAT1 inhibitor represses several AD markers expressions and accelerate the proliferation of mouse hippocampal neuronal cells. These findings might offer some explanations why the downregulated expression of STAT1 is associated with longer lifespan of AD patients in the present study. In addition, the recent study of Zhang et al. (2022) shows that pharmacological degradation and inhibition of BRD4, which affects transcriptional regulation of autophagy and lysosome genes, significantly increase Aβ levels that are related to AD neuropathology in cell models, indicating that the upregulation of BRD4 might be beneficial for AD, consistent with the findings of the present study that BRD4 was upregulated in longer-lived AD patients and corelated with multiple immune cells and factors (Figure 5).

Interestingly, the enrichment analysis *via* DisGeNET (Figures 4C,D) revealed noteworthy overlaps with neoplastic diseases in both up-and downregulated DEGs of AD comparison. Several AD-lifespan-related pathways identified in the present study were also related to cancer, such as positive regulation of cell death, Malignant pleural mesothelioma, Hippo signaling pathway in the downregulated DEGs and apoptotic cell clearance, Signaling by Receptor Tyrosine Kinases in the upregulated DEGs. These results indicated that the regulation of AD Lifespan and cancer might share common pathways. Nudelman et al. have reviewed about ten hallmark biological alterations which overlap in the pathogenesis of cancer and AD (Nudelman et al., 2019), and proposed that pathways related to inflammation might exhibit similar roles and parallel directions of regulation in the pathogenesis of cancer and AD (Nudelman et al., 2019). It has been assumed that inflammation might accelerate the earliest development of neoplastic progression, especially a chronic state of systemic inflammation. To survive, tumors need to shift the subclasses of immune cells attacking the tumor toward those promoting inflammation and tumor growth (Singh and Singh, 2015; Goswami et al., 2017). As for AD, increasing pro-inflammatory cytokine burden has been proved in AD patients’ brains. Epidemiological studies have also shown that long-term use of chronic non-steroidal anti-inflammatory drugs prior to AD onset relates to a lower incidence (Taylor et al., 2018). Thus, regulating the overlapping pathways or genes related to inflammation might be beneficial for the interventions of both cancer and LOAD.

Recent studies have shown that HDAC6 might be of dual function in the regulation of both AD and cancer. Ruzic et al.’s (2022) study discovered two HDAC6 inhibitors with anti-breast cancer activity. As for AD, HDAC6, has shown elevated levels in AD with direct interaction with the tau protein (Qureshi and Chinnathambi, 2022) while Sreenivasmurthy et al.’s (2022) study shows that inhibiting HDAC6 leads to activation of chaperone-mediated autophagy and alleviation of tau pathology in AD models. In the present study, HDAC6 was among the top 10 hub genes identified in the downregulated DEGs of longer-lived AD and corelated with multiple immune cells and factors, indicating that HDAC6 was closely associated with neuroinflammation and its downregulation might be helpful to prolong AD lifespan, concurring with previous studies.

Also, IL6 (meta *p* = 0.002, log FC = −1.01) and CD36 (meta *p* = 0.012, log FC = 1.96) might be potential therapeutic targets, both of which were involved in the pathway related to neuroinflammation in the present study. Escrig et al. study shows that the inhibition of IL-6 trans-signaling partially rescues the AD-induced mortality and reverses AD-induced cognitive and emotional changes in AD animal models, presenting strong potentials as a powerful therapeutic target in AD (Escrig et al., 2019). Interestingly, blocking IL-6 or inhibiting its associated signaling has been proposed to be a potential therapeutic strategy for the treatment of cancers with IL-6-dominated signaling (Kumari et al., 2016). As for CD36, Wang et al.’s study found that upregulating CD36 expression ameliorated hypoxia-induced neuroinflammation, diminished Aβ deposition, and improved spatial memory defects in APP/PS1 mice (Wang et al., 2014). Meanwhile, Fang et al. (2019) report about the tumor-suppressive effects of CD36 and that CD36 inhibits growth and metastasis of colorectal cancer cells *in vivo*. These findings indicate that IL6 and CD36 might exert parallel function in the regulation of both AD and cancer, serving as promising targets for the two.

To sum up, neuroinflammation might take the center stage in the regulation of AD lifespan and it might be of particular importance to uncover the pathways or genes related to inflammation, especially those exhibiting parallel directions of regulation in the pathogenesis of cancer and AD, which might be promising targets for both diseases.

## 5. Limitations

The findings of the present study must be interpreted in the light of certain limitations. Firstly, the data used in the present study were obtained from multiple studies, increasing the risk of confounding effects, such as sample size, sample sources and processing, quality and amount of RNA, microarray platform and so on. However, we tried to minimize these effects by selecting samples from the temporal lobe only and including datasets using similar techniques; we also adopted RRA for gene list integration and meta-analysis to reduce batch effects. Secondly, due to the limited number of genes exported from GEO2R when using the standard of adjust value of *p* < 0.05, *p* value < 0.05 was adopted for the first screening of DEGs, which might cause false positive results. However, after the first screening, we used RRA for value of *p* meta-analysis, which is designed to integratively select DEGs appearing in multiple datasets with high ranking. RRA has been reported to be robust and accurate in detecting DEGs across datasets. Then the results were screened for the second time using the standards of meta *p* values less than 0.05 and average |log FC|s ≥ 1 in order to further reduce false positive rate. Thirdly, since RNA-Seq technique is more powerful than microarray in evaluating gene expression profiles, thorough search and data digging were performed to locate suitable RNA-seq datasets for the present study. One such dataset was located but not included as previously mentioned. Continuous attention will be paid to newly-published studies or datasets in order to incorporate more data timely.

## 6. Conclusion

The results of the present study showed that neuroinflammation might take the center stage in the regulation of AD lifespan and it might be of particular importance to uncover the pathways or genes related to inflammation, especially those exhibiting parallel directions of regulation in the pathogenesis of cancer and AD, which might be promising targets for both diseases. The involved pathways and genes identified in the present study might provide information about lifespan-related genetic mechanisms in AD patients and help developing promising strategies in further investigation.

## Data availability statement

The data presented in the study are deposited in the Gene Expression Omnibus (GEO, http://www.ncbi.nlm.nih.gov/geo/) repository, accession numbers: GSE48350, GSE5281, GSE28146, GSE36980.

## Author contributions

FY was responsible for the rationale and the design of the study and edited and approved the final manuscript. JZ and YX conducted the series of dataset search, data processing, and relevant bioinformatics analyses. JZ wrote the manuscript. XL and JX assisted with the data analyses and manuscript editing, respectively. All authors contributed to the article and approved the submitted version.

## Funding

The authors disclosed receipt of the following financial support for the research, authorship, and/or publication of this article: The study was carried out in Chengdu, China, and funded by Clinical Research and Translational Foundation of Sichuan Provincial People’s Hospital (2021LY11).

## Conflict of interest

The authors declare that the research was conducted in the absence of any commercial or financial relationships that could be construed as a potential conflict of interest.

## Publisher’s note

All claims expressed in this article are solely those of the authors and do not necessarily represent those of their affiliated organizations, or those of the publisher, the editors and the reviewers. Any product that may be evaluated in this article, or claim that may be made by its manufacturer, is not guaranteed or endorsed by the publisher.

## Footnotes

^1^ http://www.ncbi.nlm.nih.gov/geo/

^2^ http://metascape.org

^3^ http://cis.hku.hk/TISIDB/download.php
